# Impact of A1 segment asymmetry on hemodynamic conditions around the circle of Willis and anterior communicating artery aneurysm formation

**DOI:** 10.3389/fneur.2024.1491247

**Published:** 2025-01-07

**Authors:** Joonho Lee, Seul-Ki Jeong, Ji Man Hong

**Affiliations:** ^1^Department of Neurology, Ajou University School of Medicine, Suwon, Republic of Korea; ^2^Seul-Ki Jeong Neurology Clinic, Seoul, Republic of Korea; ^3^Department of Convergence of Healthcare and Medicine, Ajou University Graduate School of Medicine, Suwon, Republic of Korea

**Keywords:** circle of Willis (CoW), anterior communicating aneurysm, A1 asymmetry, signal intensity gradient, shear stress

## Abstract

**Background:**

This study aims to investigate how A1 segment asymmetry—also known as A1 dominancy—influences the development of the anterior communicating artery aneurysm (AcomA) as it affects hemodynamic conditions within the circle of Willis (COW). Using time-of-flight magnetic resonance angiography (TOF-MRA), the research introduces a novel approach to assessing shear stress in A1 segments to uncover the hemodynamic factors contributing to AcomA formation.

**Method:**

An observational study was conducted over 6 years at a tertiary university hospital’s outpatient clinic. Recruited patients who underwent TOF-MRA imaging were divided into AcomA and non-AcomA groups. MRA images were analyzed using semi-automatic software (VINT, Mediimg, Inc.) to calculate the signal intensity gradient (SIG), which reflects wall shear stress. The comparison metrics included general demographics, anatomical characteristics, and hemodynamic attributes of the COW, mainly focusing on A1 segment asymmetry.

**Results:**

Among the 700 subjects, 106 were categorized into the AcomA group, while 594 were placed in the non-AcomA group. The AcomA group showed a more significant difference in the bilateral A1 diameter (49.0% vs. 20.8%, *p* < 0.001) and a greater prevalence of unilateral A1 aplasia (32.1% vs. 6.7%, *p* < 0.001) compared to the non-AcomA group. Increased bilateral A1 asymmetry in the AcomA group corresponded with notable variations in A1 SIG, indicating increased wall shear stress. The occurrence of AcomA is associated with both anatomical factors of the circle of Willis, represented by the bilateral A1 diameter ratio, and hemodynamic factors, represented by the bilateral A1 SIG ratio, suggesting that both factors are almost equally significant.

**Conclusion:**

Our findings suggest that A1 segment asymmetry influences hemodynamic changes within the COW, contributing to AcomA formation. Hemodynamic factors provide an intuitive understanding of how anatomical characteristics within the COW can lead to aneurysm development.

## Introduction

It has been recognized that intracranial artery aneurysms primarily occur around the circle of Willis (COW), and that the diversity of COW structure is related to the occurrence of intracranial artery aneurysm (1). Several studies have focused on the anterior communicating artery due to its frequent occurrence and rupture of aneurysms in this location, hypothesizing that the inequality in bilateral A1 is linked to the occurrence of anterior communicating artery aneurysm (AcomA) (2–5). Using CT angiography or transcranial color-coded sonography, it has been demonstrated that bilateral A1 inequality is associated with aneurysm occurrence, with the difference in radius between bilateral A1, termed A1 dominance, being particularly significant (4–6). However, there is still no consensus on criteria and standard methods for measuring hemodynamic stress derived from this inequality, as only a limited number of studies have been conducted.

Wall shear stress (WSS) refers to the force applied to the tube wall by fluid movement, primarily generated *in vivo* by blood flow, exerting force on the endothelium and inducing structural and biochemical changes (7). Consequently, it has been suggested that WSS may be linked to vascular disease occurrence, with various studies investigating the relationship between cerebral aneurysms and WSS. It has been observed that high WSS is applied to bifurcation sites, where intracranial artery aneurysms frequently occur, and changes induced by high wall shear stress are associated with aneurysm occurrence, with WSS decreasing as the aneurysm grows (8–10). Recent studies have revealed that the anatomical diversity of the COW and its surrounding vessels influences the distribution of hemodynamic factors, such as wall shear stress in the vasculature (11). However, as mentioned earlier, while previous studies have demonstrated that the COW’s anatomy is associated with the occurrence of intracranial artery aneurysms, investigations specifically addressing the hemodynamic characteristics of the COW, such as shear stress, have been relatively rare. Recently, additional research efforts have been made (12), though the causal relationship remains a subject of ongoing debate (13).

Given the association between shear stress and aneurysm occurrence and the relationship between bilateral A1 inequality and AcomA occurrence, we utilized a novel method using magnetic resonance angiography (MRA) to obtain shear stress applied to the vessel wall. We then analyzed the association between A1 dominance in AcomA patients and bilateral A1 wall shear stress.

## Methods

### Study population

**Figure 1:**
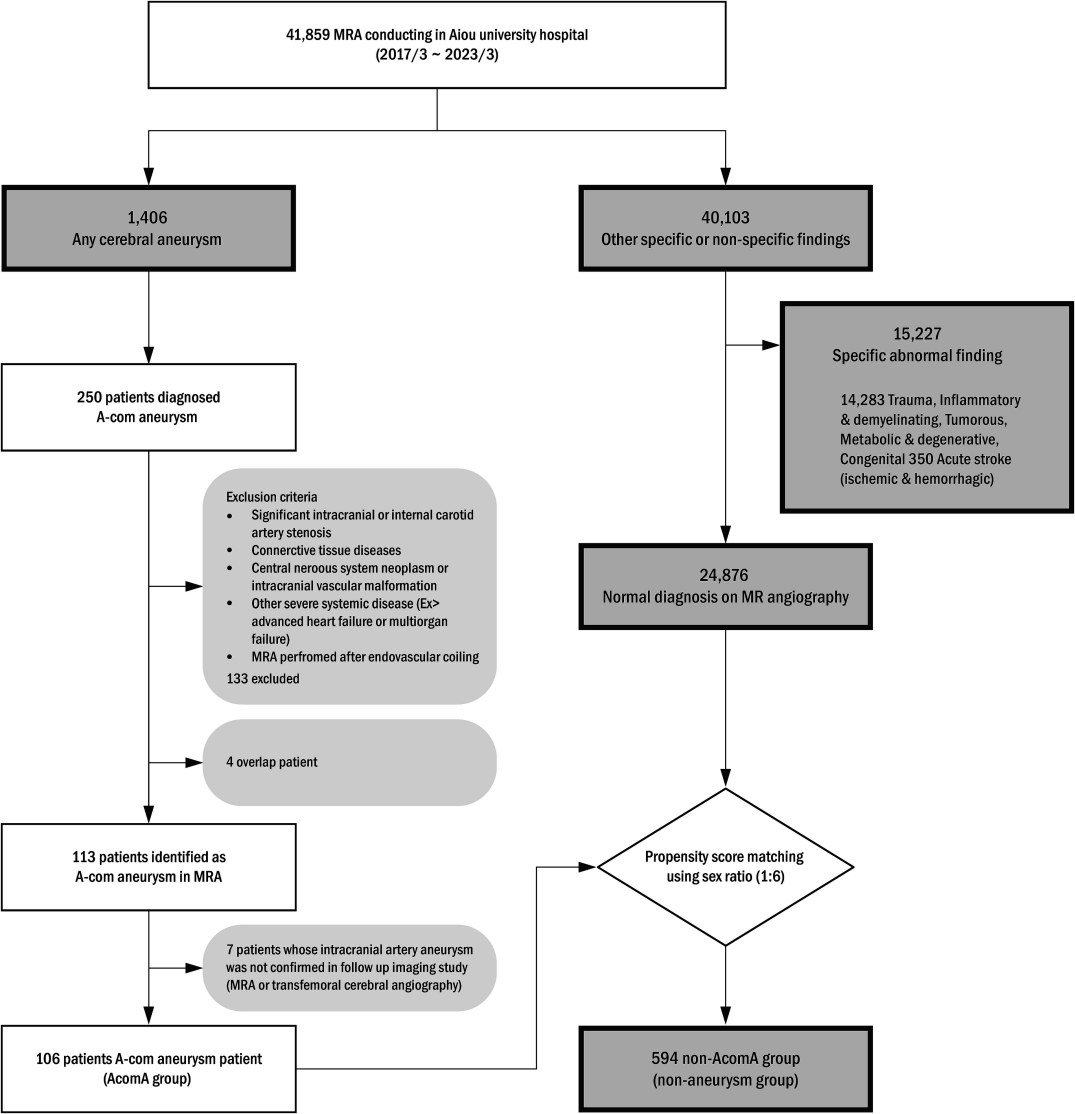
Anterior communicating artery (A-com) aneurysm patient selection algorithm. MRA, magnetic resonance angiography.

For this study, we retrospectively collected data from adult patients who had undergone magnetic resonance angiography (MRA) and were diagnosed with intracranial artery aneurysms based on the medical records of the period between January 2017 and March 2023 from a tertiary hospital. Among them, patients with radiologically confirmed AcomA on MRA imaging were selected. The exclusion criteria of the study were determined with (1) coexistence of significant intracranial or internal carotid artery stenosis, (2) connective tissue diseases that can affect the development of intracranial artery aneurysm, (3) central nervous system neoplasm or intracranial vascular malformation, (4) other severe systemic diseases, such as advanced congestive heart failure or multiorgan failure, (5) cases in which MRA achievement was performed after endovascular coiling. From the initial cohort, 250 AcomA candidates were identified as the above eligible criteria, and 137 candidates were deemed ineligible. A subsequent seven candidates were excluded due to inconclusive results on their follow-up MRA or transfemoral angiography. Eventually, 106 patients were included in the study. For a comparison analysis, a distinct control cohort was constituted from out-patient clinic patients who performed an image including MRA. Within this control cohort, abnormal findings observed on MRI were excluded, propensity score matching using gender was performed, and 594 individuals were finally selected to serve as a control group. The entire patient selection process and methodology are illustrated in Figure 1. Comprehensive patient profiles were recruited, encompassing clinical information such as hypertension (HTN), diabetes mellitus (DM), hyperlipidemia, and historical data on smoking habits, cardiac diseases, and laboratory test findings. This study was approved by the Ajou University Institutional Review Board.

### Classification method of anterior cerebral circulation anatomic variants

**Figure 2:**
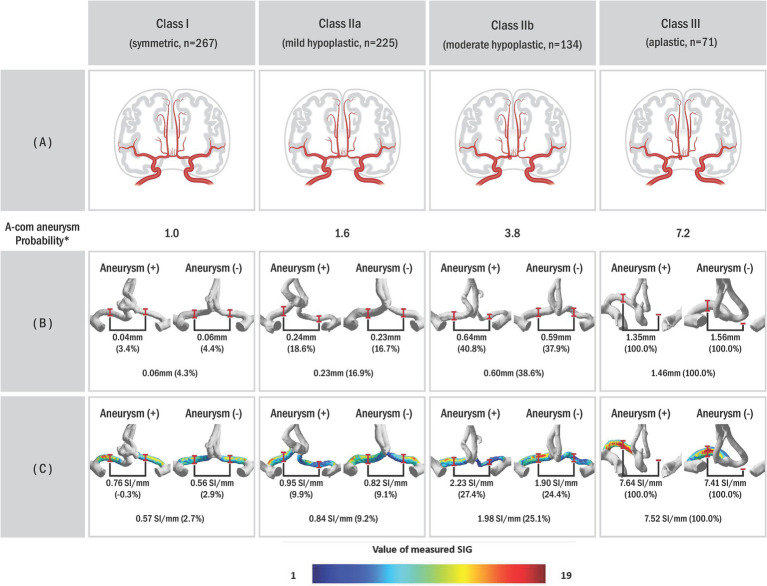
The probability of AcomA occurrence according to the circle of Willis (COW) types. **(A)** Schematic illustrations of anterior communicating artery anatomic variations with aneurysm, **(B)** anterior cerebral circulation three-dimensional (3D) model of actual patient reconstructed through the program, and **(C)** visualized A1 signal intensity gradient of 3D model. In our study, we classified subjects into different categories based on a classification system where Class I represented vascular asymmetry index (VAI) less than 10%, Class II represented VAI of 10% or higher, subdivided into Class IIa (VAI < 25%) and Class IIb (VAI ≥ 25%), and Class III indicated unilateral A1 aplastic type on the 3D model. *Probability was calculated based on the odds ratio of Class I. The odds ratio can be obtained using the ratio of each type according to the presence or absence of an A-com aneurysm.

Each anatomical variation type was defined to understand the anatomical variance of the circle of Willis based on the presence or absence of aneurysms (Figure 2). If the radius between bilateral sides differed by less than 10%, it was categorized as the bilateral A1 symmetric type (Class I). If the difference was 10% or more, it was classified as the unilateral A1 hypoplastic type (Class II). Among the unilateral A1 hypoplastic type, if the radius difference was less than 25%, it was considered mild (Class IIa). If the difference was 25% or more, it was considered moderate (Class IIb). This subdivision aimed to balance the sample sizes and better observe the trend of increasing A-com aneurysm occurrence with more significant asymmetry. The unilateral A1 aplastic type (Class III) was determined as a case where one A1 segment was not identified in the 3D reconstruction model. The probability of A-com aneurysm occurrence was calculated using the A-com aneurysm odds ratio according to each anatomy type. The vascular asymmetry index (VAI, %) was defined as the ratio of the bilateral A1 radius difference to the dominant A1 radius, expressed as (1 − non-dominant A1 radius/dominant A1 radius) × 100, to indicate the difference in the bilateral A1 radius. The SIG difference ratio (%) was also defined as (1 − non-dominant A1 SIG/dominant A1 SIG) × 100.

### Wall shear stress analysis

**Figure 3:**
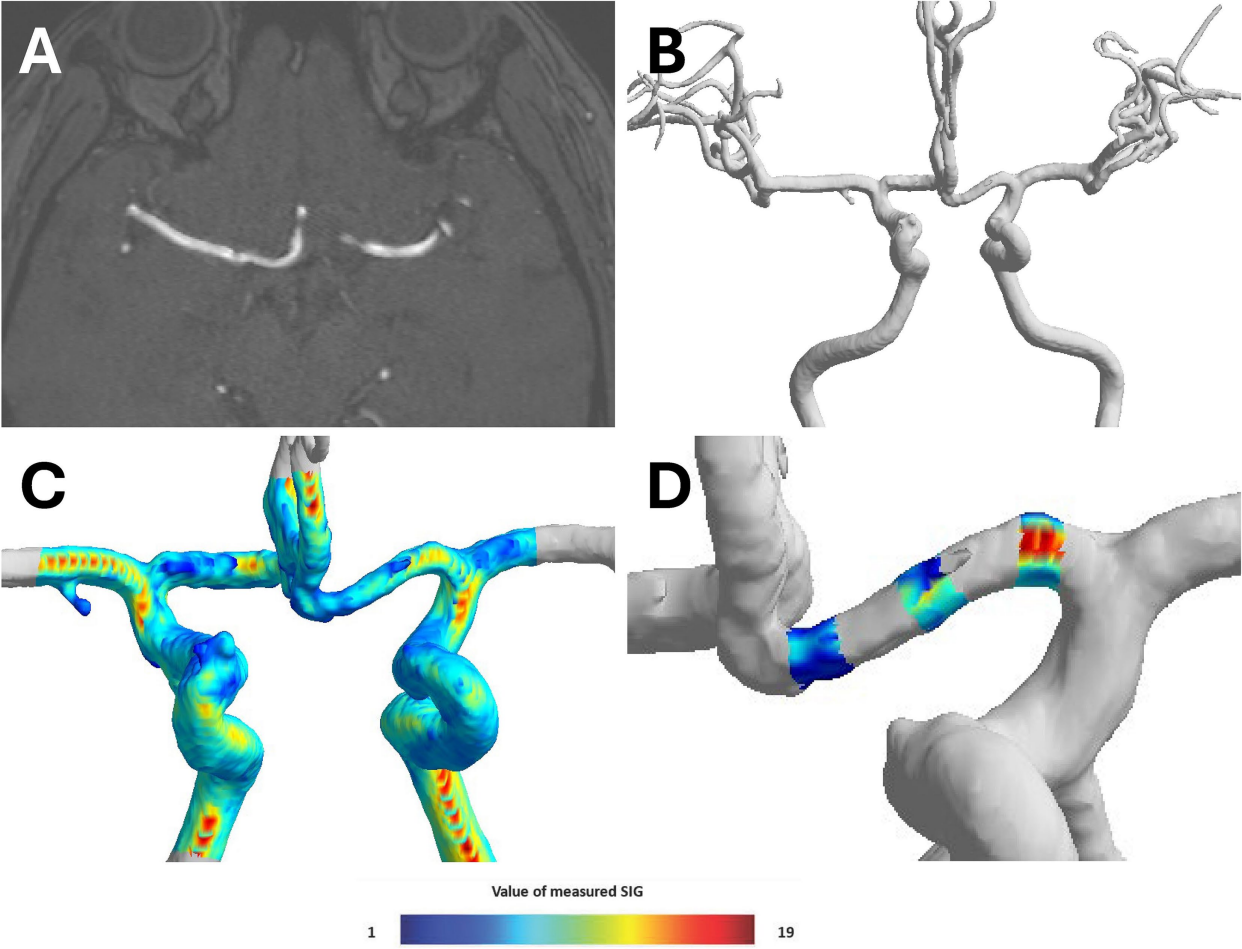
Process of converting TOF-MRA image to three-dimensional (3D) model. **(A)** Time-of-flight magnetic resonance angiography (TOF-MRA) coronal image of brain, **(B)** reconstructing three-dimensional (3D)-model of cerebral anterior circulation by semi-automated software (VINT, Mediimg, Inc.), **(C)** displaying signal intensity gradient (SIG), which represent shear stress, on 3D-models using color, and **(D)** three locations (proximal, middle, and distal) of 3D model A1 segment were determined, and radius and SIG value were measured in cross sections perpendicular to each axis.

For this study, we used semi-automated software (VINT, Mediimg, Inc.) to reconstruct a time-of-flight (TOF) sequence obtained from magnetic resonance imaging (MRI) (Figure 3A) for wall shear stress analysis. As a result of this program analysis, the arterial wall signal intensity gradient (SIG) was obtained. Arterial wall SIG conceptually corresponds to shear rate (or velocity gradient) and has been validated for wall shear stress from computational fluid dynamics (14) and phase-contrast magnetic resonance (15). In addition, the three-dimensional (3D) model of the intracranial artery was created with the software (Figure 3B), and the measured SIG value was reflected in color on the 3D model (Figure 3C).

**Figure 4:**
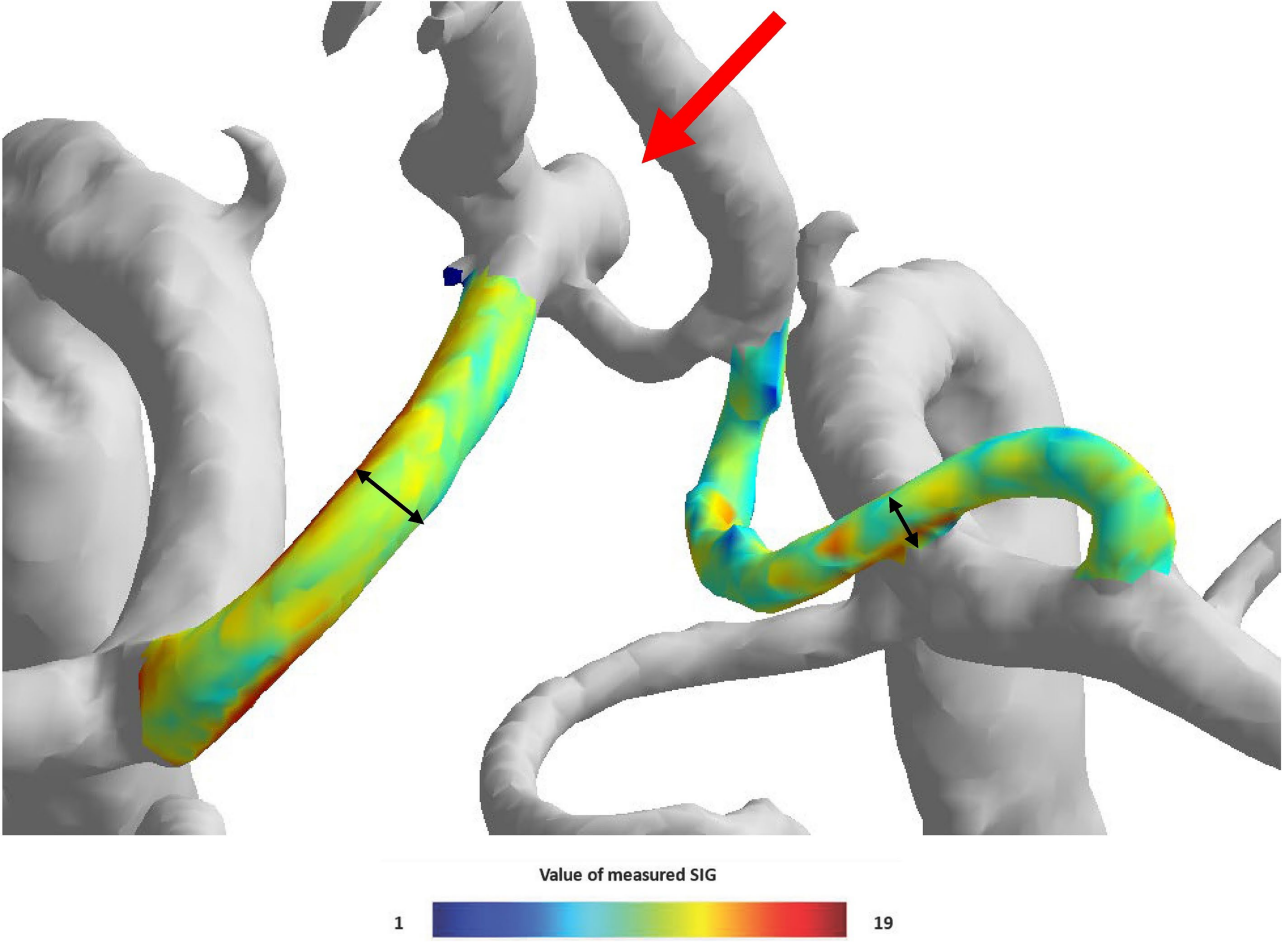
The visual appearance of the left side anterior communicating artery aneurysm and both A1 signal intensity gradient (SIG) in the three-dimensional (3D) model.

The TOF-MRA source image was extracted and used as Digital Imaging and Communications in Medicine (DICOM) files. The extracted source image was reconstructed using the software to create intracranial artery system 3D model. To analyze bilateral A1 artery in 3D model, only anterior cerebral circulation was first separated. Three locations (proximal, middle, and distal) of 3D model A1 segment were determined, and radius and SIG value were measured in cross-sections perpendicular to each axis. Furthermore, A1 segment was analyzed by mean of measured radius and SIG values (Figure 3D). The direction of AcomA was chosen after the researcher identified the remodeling 3D model (Figure 4).

### Statistical analysis

All analyses were performed using R studio software version 1.4 (R Foundation for Statistical Computing, Vienna, Austria). The results of the analyses were reported as numbers (percentages), means ± standard deviation (SD), or medians (interquartile ranges [IQRs]). First, it was checked whether the data satisfies normal distribution. For continuous variables comparison, the Student’s *t*-test was used if regular analysis was satisfied, and the Wilcox test was used if not satisfied. Pearson’s chi-squared test or Fisher’s exact test was used when comparing category variables.

It is known that gender and aneurysm occurrence are related (16). Still, when the control group was selected at random, the sex ratio of the control group was significantly different from that of the patient group, so propensity score matching using sex ratio was performed. The propensity score was calculated with the multivariable logistic regression model using demographics. We matched AcomA group with non-AcomA group in a 1:6 ratio with nearest neighbor matching, incorporating gender as a covariate. Finally, 106 AcomA groups and 594 non-AcomA groups were used for analysis. The standardized mean difference was 0.07, which was judged to have been appropriately matched.

Pearson’s correlation test was used to analyze the correlation between variables. Univariate analysis was used to determine the significant variables for multivariate logistic regression models. *p* < 0.05 was considered statistically significant for every analysis.

## Results

### Demographic characteristics

Notably, 106 groups with aneurysms and 594 groups without aneurysms as control groups were classified in the study (Figure 1). The characteristics of the study population are summarized in Table 1. No significant age (mean 61.2 vs. 59.3, *p* = 0.055) and sex (male percentage; 43.4% vs. 49.6%, *p* = 0.878) difference between the two groups was confirmed. When checking the underlying clinical and social history, hypertension (54.9% vs. 30.7%, *p* < 0.001) shows a significant difference, but no other specific results were identified. All laboratory testing results showed a significant difference between the two groups except for high-density lipoprotein and total cholesterol. When checking the underlying clinical and social history, hypertension (54.7% vs. 29.8%, *p* < 0.001), diabetes (20.8% vs. 12.0%, *p* = 0.002) and coronary artery occlusive disease (9.43% vs. 1.68%, *p* < 0.001) show a significant difference, but no other specific results were identified. When comparing the two groups, the laboratory results showed significant differences in serum glucose (115.7 mg/dL vs. 101.2 mg/dL, *p* < 0.001), hematocrit (40.4 vs. 43.3, *p* < 0.001), HDL (52.5 mg/dL vs. 59.7 mg/dL, *p* = 0.002), LDL (90.4 mg/dL vs. 115.2 mg/dL, *p* < 0.001), and total cholesterol (175.3 mg/dL vs. 196.8 mg/dL, *p* < 0.001) in both groups.

**Table 1:** Demographics, A1 anatomy type, and hemodynamic characteristics of groups with and without aneurysm.

	AcomA group (*n* = 106)	Non-AcomA group (*n* = 594)	*p*
Demographic characteristics			
Sex (male %)	46 (43.4)	253 (49.6)	0.878
Age (years)	61.2 ± 10.9	59.3 ± 8.7	0.055
Underlying disease			
Hypertension, *n* (%)	58 (54.7)	177 (29.8)	<0.001
Diabetes, *n* (%)	22 (20.8)	71 (11.9)	0.002
CAOD, *n* (%)	10 (9.4)	10 (1.7)	<0.001
BMI (kg/ $m^{2}$ )	24.6 ± 3.4	24.35 ± 3.1	0.802
Smoking history, *n* (%)	19 (17.9)	137 (23.1)	0.268
Baseline laboratory findings			
Serum glucose (mg/dL)	115.7 ± 34.8	101.2 ± 19.9	<0.001
Hematocrit (%)	40.4 ± 4.6	43.3 ± 4.2	<0.001
HDL (mg/dL)	52.5 ± 14.9	59.7 ± 26.4	0.002
LDL (mg/dL)	90.4 ± 33.9	115.2 ± 37.9	<0.001
Total cholesterol (mg/dL)	175.3 ± 39.8	196.8 ± 41.2	<0.001
Willisian anatomy around A1, *n*			
Class I (bilateral A1 symmetric, VAI <10%)	17 (16.0)	250 (42.1)	
Class II (VAI ≥ 10%)	55 (51.9)	304 (51.2)	
Class IIa (mild, 25% > VAI ≥ 10%)	23 (21.7)	202 (34.0)	
Class IIb (moderate, VAI ≥ 25%)	32 (30.2)	102 (17.2)	
Class III (unilateral A1 aplastic)	34 (32.1)	40 (6.7)	
A1 anatomy characteristics			
Dominant A1 radius (mm)	1.4 ± 0.4	1.40 ± 0.2	0.193
Non-dominant A1 radius (mm)	0.7 ± 0.5	1.09 ± 0.3	<0.001
Bilateral A1 radius difference (mm)	0.7 ± 0.6	0.31 ± 0.4	<0.001
VAI (%)	48.9	20.8	<0.001
A1 hemodynamics on TOF-MRA			
Dominant artery SIG (SI/mm)	7.76 ± 1.45	7.27 ± 0.84	0.001
Non-dominant SIG (SI/mm)	4.45 ± 3.30	6.12 ± 1.95	<0.001
Bilateral A1 SIG difference (SI/mm)	3.45 ± 3.22	1.34 ± 1.83	<0.001
SIG difference ratio (%)	42.45	15.27	<0.001

CAOD, coronary artery occlusive disease; BMI, body mass index; HDL, high-density lipoprotein; LDL, low-density lipoprotein; VAI, vascular asymmetry index; SIG, signal intensity gradient.

### Anatomic variants of anterior cerebral circulation

Table 1 also shows that the A1 anatomy significantly differs between the two groups. In the group with aneurysm, there were fewer Class I (16.0% vs. 42.1%) and more Class III (32.1% vs. 6.7%). Furthermore, Figure 4 illustrates that as the imbalance of bilateral A1 increases, the probability of the anatomic variation type, depending on the presence or absence of an aneurysm, also increases.

As previously mentioned, AcomA typically occurs at the bifurcation of A2 and A-com, often in the direction of the dominant artery. Out of 89 identified patient aneurysm directions on the 3D reconstruction model (except when the aneurysm was too large to discern the direction, *n* = 17), 75 occurred in the direction of the dominant artery (Table 2). The chi-square test demonstrated a relationship between the anatomy type and the direction of AcomA occurrence. As the degree of bilateral A1 asymmetry increases, there is a tendency for the occurrence rate to be higher in the direction of the dominant A1.

**Table 2:** The directionality of AcomA based on the anatomical characteristics of A1 segments in the AcomA group (*n* = 106).

Anatomical asymmetry features, *n* (%)	A1 dominant (*n* = 75)	A1 non-dominant (*n* = 14)	Not categorized (*n* = 17)	*p (dominant vs. non-dominant)
Class I (symmetric, VAI < 10%)	7 (9.3%)	8 (57.1%)	2	0.796
Class IIa (mild, 25% > VAI ≥ 10%)	13 (17.3%)	2 (14.3%)	8	0.005
Class IIb (moderate, VAI ≥ 25%)	24 (32.0%)	3 (21.4%)	5	<0.001
Class III (unilateral A1 aplastic)	31 (41.3%)	1 (7.1%)	2	<0.001

VAI, vascular asymmetry index.

*By chi-squared goodness of fit test, not categorized group is excluded.

### Hemodynamics stress

Comparing the measured radius values, the group with aneurysms exhibited significantly smaller values in the non-dominant artery (0.67 mm vs. 1.09 mm, *p* < 0.001). In contrast, the dominant artery showed no significant difference (1.36 mm vs. 1.40 mm, *p* = 0.193) between the two groups. Regarding SIG values, there was a substantial difference in both the dominant artery (7.76 SI/mm vs. 7.27 SI/mm, *p* = 0.001) and the non-dominant artery (4.45 SI/mm vs. 6.12 SI/mm, *p* < 0.001). Additionally, the two groups the two groups had a significant SIG difference (3.55 SI/mm vs. 1.48 SI/mm, *p* < 0.001).

The VAI was larger in the group with the aneurysm, indicating that the difference in dominant and non-dominant A1 radius in the group with aneurysms was significantly larger (49.0% vs. 20.8%, *p* < 0.001). Similarly, the SIG difference ratio was larger in the group with aneurysms (42.5% vs. 15.3%, *p* < 0.001). When analyzed according to the anatomy type, it was found that the larger the difference between bilateral A1 radii, the larger the difference between bilateral A1 SIG values (Figure 4C).

**Figure 5:**
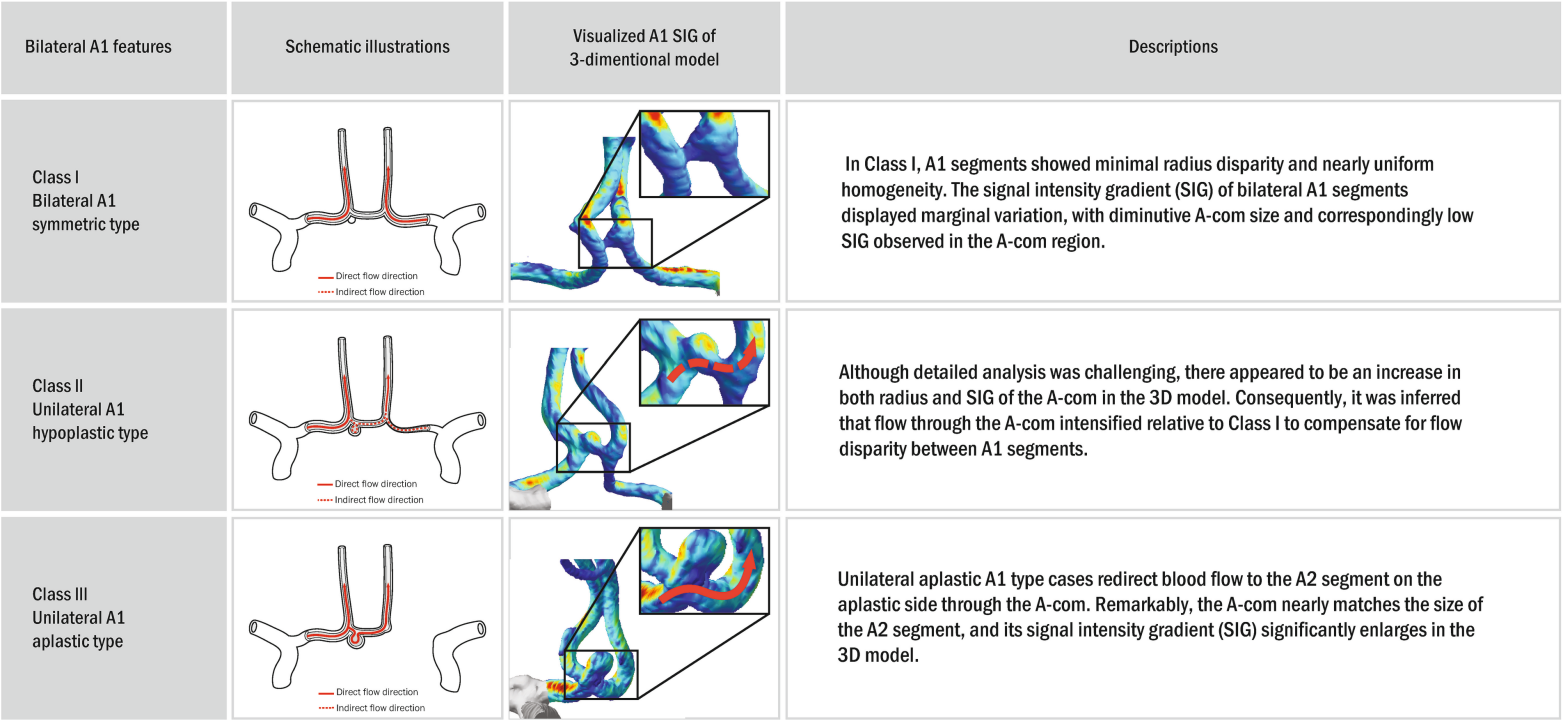
Schematic illustrations and visualized anterior circulation SIG on a three-dimensional (3D) model. In the three-dimensional (3D) model, the bilateral A1 symmetric type (Class I) exhibited no definite disparity in diameter or wall shear stress between A1 and A2. Furthermore, the dimensions and wall shear stress of the A-com were modest. However, with increasing asymmetry, unilateral A1 hypoplastic type (Class II) a trend toward enlargement of the A-com diameter and a concomitant elevation in wall shear stress were observed. In the unilateral A1 aplastic type (Class III), characterized by A-com providing arterial supply to all A2 branches on the aplastic side, the A-com appeared commensurate in size with A2, accompanied by a notable increase in wall shear stress. Nonetheless, this tendency has not been statistically proven.

As asymmetry increased, the diameter of A-com tended to increase, and wall shear stress also appeared to increase (Figure 5). In Class III, where A-com supplies all A2 on the aplastic side, A-com is as large as A2, and wall shear stress also appears large. However, this tendency has not been statistically proven.

### Multivariate analysis to predict anterior communicating artery aneurysm

**Figure 6:**
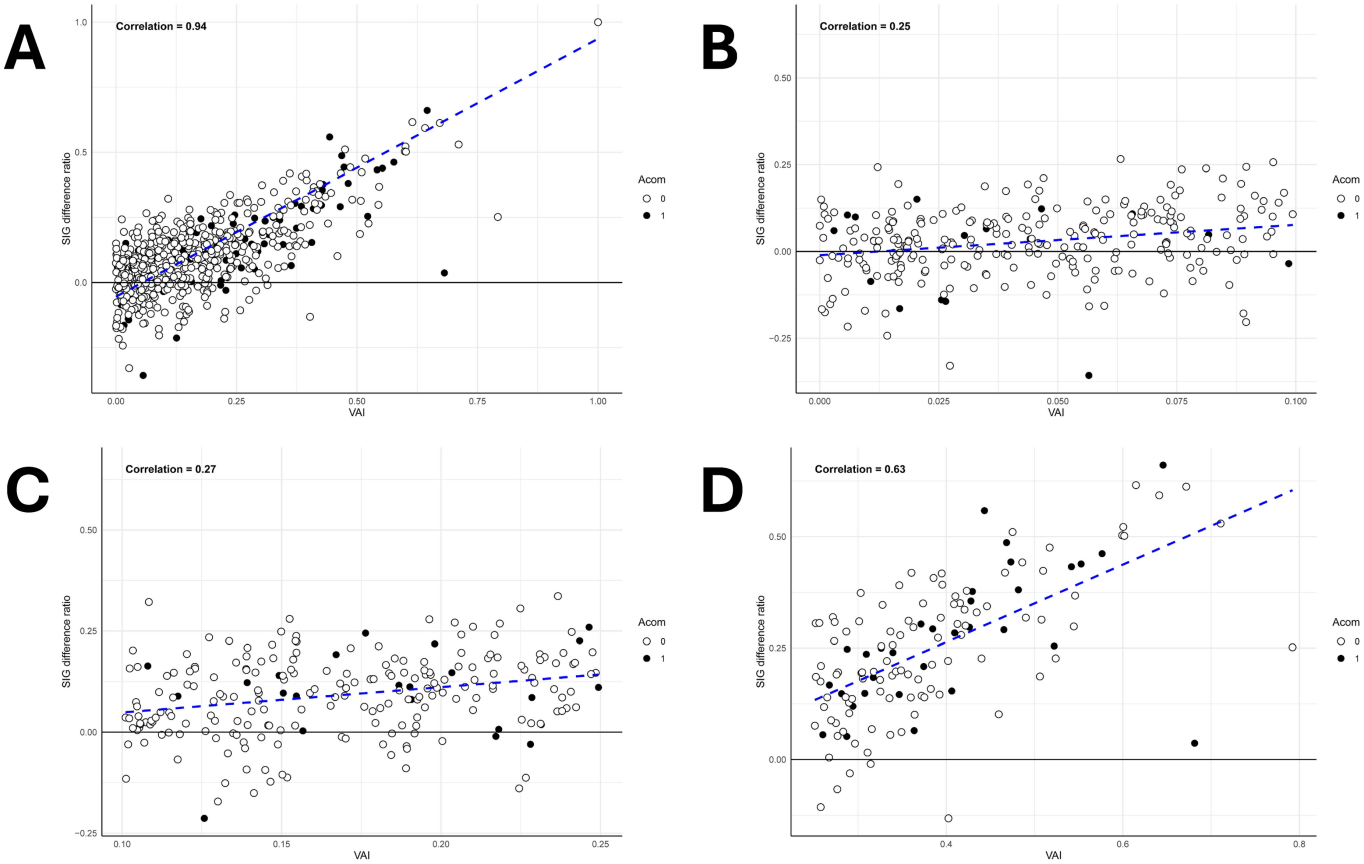
Scatter plot analysis of vascular asymmetry index (VAI) and signal intensity gradient (SIG) difference ratio across A1 morphological classifications. **(A)** The scatter plot graph between the SIG difference ratio and the VAI shows a high correlation with a correlation coefficient of 0.94. **(B)** The scatter plot graph of the bilateral A1 symmetric type (Class I) which shows a correlation coefficient of 0.25. **(C)** The scatter plot graph of the mild unilateral A1 hypoplastic type (Class IIa) which shows a correlation coefficient of 0.27. **(D)** The scatter plot graph of the unilateral A1 hypoplastic type (Class IIb) which shows a correlation coefficient of 0.63.

The VAI and SIG difference ratio exhibited a very high correlation with a correlation coefficient, 0.94 (Figure 6). Among them, excluding Class III, Class I (symmetric) showed a correlation coefficient of 0.16, indicating little correlation. In contrast, Class IIa (mild asymmetric) showed a higher correlation of 0.28, and Class IIb (moderate asymmetric) showed an even higher correlation of 0.60. Therefore, it was observed that the correlation coefficient between VAI and SIG difference ratio increased as the asymmetry of bilateral A1 increased.

The variables with significant results in univariate analysis are presented in Table 3. Significant factors were included in multiple regression analysis to predict the occurrence of AcomA. Given the high correlation among the SIG difference ratio, VAI, and A1 radius difference, hierarchical regression, and stepwise regression were used to identify the most influential factors on AcomA aneurysm occurrence. However, when these three factors were included, none were statistically significant, likely due to high multicollinearity. Additionally, when included separately, there was no significant difference in model fit: the AIC values were 451.8 for the SIG difference ratio, 452.2 for the A1 radius difference, and 439.3 for VAI. The SIG difference ratio was chosen for further analysis because it indicates wall shear stress. The multiple regression analysis revealed that hypertension, diabetes, coronary artery occlusive disease, serum glucose, hemoglobin, HDL, LDL, total cholesterol, and SIG difference ratio were significant. However, among these, diabetes, HDL, LDL, and total cholesterol had odds ratios close to 1. In contrast, the odds ratio of SIG difference ratio was significantly higher, at approximately 9.61, indicating a stronger association with the condition. We applied the backward elimination technique to determine whether a model with a lower AIC value could be identified; however, the model including all the variables had the lowest AIC value. Nevertheless, when comparing models with individual variables removed, the model without VAI showed a relatively significant increase in the AIC value to 493.6, confirming that VAI is the most important variable among those analyzed.

**Table 3:** Univariate and multivariate analysis of variables associated with the occurrence of anterior communicating artery aneurysm.

	Univariate analysis			Multivariate analysis		
	95% CI	Crude OR	*p*	95% CI	Adjusted OR	*p*
Hypertension	1.86–4.32	2.83	<0.001	1.17–3.28	1.96	0.034
Diabetes	1.09–3.16	1.88	0.019	0.19–0.91	0.43	0.010
CAOD	2.42–15.10	6.04 0.07	<0.001	1.29–14.06	4.30	0.034
BMI	0.95–1.09	1.02	0.560	–	–	–
Smoking history	0.42–1.24	0.74	0.270	–	–	–
Serum glucose	1.01–1.03	1.02	<0.001	1.01–1.03	1.02	<0.001
Hemoglobin	0.81–0.90	0.86	<0.001	0.79–0.89	0.84	<0.001
HDL	0.96–0.99	0.97	<0.001	0.94–0.98	0.96	<0.001
LDL	0.97–0.99	0.98	<0.001	0.94–0.98	0.96	<0.001
Total cholesterol	0.98–0.99	0.99	<0.001	1.02–1.05	1.03	<0.001
A1 radius difference*	2.71–5.88	3.98	<0.001	–	–	–
VAI*	7.10-23.78	12.93	<0.001	–	–	–
SIG difference ratio*	5.19–16.04	9.09	<0.001	4.93–18.96	9.61	<0.001

*These three factors had a high correlation and neither factor was particularly dominant.

CAOD, coronary artery occlusive disease; BMI, body mass index; HDL, high-density lipoprotein; LDL, low-density lipoprotein; A1 radius difference, the difference in radius between the bilateral A1 segments; VAI, vascular asymmetry index is defined as [1 − (dominant A1 radius/non-dominant A1 radius)] × 100; SIG difference ratio defined as [1 − (dominant A1 SIG/non-dominant A1 SIG)] × 100.

## Discussion

In this study, we identified significant differences in the anatomical dominance of the A1 segment within the Willisian circle, based on the presence or absence of the anterior communicating artery aneurysm, seamlessly consistent with previous anatomy-driven investigations. Moreover, by employing the TOF-MRA technique for a cutting-edge hemodynamic analysis of a scalar mode, we observed that as the asymmetry between the bilateral A1 segments heightened, the AcomA group exhibited a widening SIG difference, which means the difference in shear stress between the bilateral A1 segments. This data offers a quantitative insight into the pronounced impact of hemodynamic stress on AcomA formation within the intracranial arteries surrounding the Willisian circle.

In this study, we used the new program to analyze and rebuild the TOF-MRA sequence. Flow velocity was used in most previous studies to analyze hemodynamic stress of blood vessels. Accordingly, transcranial Doppler sonography or phase-contrast MR was mainly used to measure flow velocity (4, 5). However, the Doppler ultrasound may not have a constant result depending on the measurement location or the person performing the ultrasound. In the case of phase-contrast MR, it was rarely clinically performed due to the long practice time, therefore there was an obstacle to collecting patient or control groups. TOF-MRA may stand out as a consistent and widely used technique, making it an ideal choice for analyzing observational data, irrespective of the examiner. Additionally, the VINT program significantly enhances efficiency, enabling rapid image analysis—approximately 1 min per person—to assess the Signal Intensity Gradient (SIG) results. This expedited process has facilitated recent research efforts, including studies on Moyamoya vessels and ruptured aneurysms, where comparisons of shear stress are conducted using TOF-MRA in conjunction with the VINT program (17, 18). Furthermore, as illustrated in the figures presented in this paper, the ability to visualize the intensity of shear stress through color coding can aid in the intuitive understanding of healthcare providers and patients.

Paul et al., using computed tomographic angiography, identified 10.6% unilateral A1 aplastic types among A-com artery variants (19). Our findings closely align with this study: 6.7% aplastic type (Class III) in the non-aneurysm group and 32.1% in the aneurysm group. This consistency suggests that anatomical variations influence aneurysm occurrence. Figure 4A illustrates that Willisian anatomy’s likelihood increases with bilateral A1 asymmetry, indicating a correlation with AcomA onset.

Multivariate analysis showed that radius and shear stress were significant individually, but not together, likely due to their strong correlation. Studies by Kaspera et al. and Kwon et al. confirm a larger dominant A1 radius in aneurysm patients and varying flow velocities between dominant and non-dominant A1 segments (5, 20). These findings illustrate the relationship between anatomical and hemodynamic factors. Poiseuille’s law suggests that, in ideal conditions, shear stress is inversely related to radius and directly related to blood flow, so we initially hypothesized that shear stress and radius would be correlated in some manner (2, 21). However, because the COW is an environment where ideal conditions are challenging to apply, we thought that measuring hemodynamic factors, rather than anatomical factors, might be statistically significant in predicting AcomA occurrence. In our analysis, we emphasized the SIG difference ratio to highlight hemodynamic aspects in Table 3. Interestingly, the findings showed no substantial difference between the impact of shear stress and radius, suggesting both factors have a strong relationship and ultimately exert a similar influence on AcomA formation. Although our initial hypothesis that measuring shear stress would be more beneficial than measuring radius in predicting AcomA occurrence was not confirmed, this positive correlation between shear stress and A1 radius (Figure 6) in our study adds a new dimension to understanding hemodynamic factors in the circle of Willis. Additionally, in cases where the curvature or size of A1 is inconsistent in imaging, making radius difficult to evaluate intuitively, the measurement of shear stress using programs such as the VINT program could provide additional insights for assessing AcomA occurrence in the future.

Ujiie et al. constructed a cerebrovascular model using glass tubes, revealing increased A-com flow as one side’s diameter shrank (22). Our patient data also appears to reflect similar findings in certain aspects. Table 1 highlights that the non-dominant A1’s radius and SIG disparities are more pronounced than the dominant A1’s between AcomA and non-AcomA groups. Such discrepancies suggest that a reduced non-dominant A1 size may prompt A-com flow alterations, potentially influencing AcomA development by affecting wall shear stress (WSS). In our patient images, a smaller non-dominant A1 was associated with a more pronounced A-com radius and SIG (Figure 5). Furthermore, a study performed using plastic models of the circle of Willis constructed from the MRA data of five patients also supports this inference, showing that when the bilateral A1 size is asymmetric, major part of the blood flow is supplied in the direction of the dominant A1 (12). However, accurate A-com analysis was limited due to its minute size and MRA image resolution. Future high-resolution imaging could confirm these insights.

The bilateral A1 imbalance appeared to affect not only the occurrence of AcomA but also the direction. As can be seen from Table 2, as the bilateral A1 difference increased, aneurysms tended to exist in the direction of Dominant A1. In previous studies, AcomA occurred in 70 out of 77 in the dominant A1 direction (5). Since cerebral aneurysm frequently occurs in bifurcation with considerable shear stress (8), it can be estimated that it occurs when A-com blood flow is generated from dominant A1 to non-dominant A1 and shear stress is applied to bifurcation in dominant A1 direction. As mentioned earlier, the study (12) using plastic models based on MRA images also supports this estimation, showing that when there is a significant size difference between the A1 segments, major part of the flow originates from the dominant A1. Since the researcher judged the direction as a subjective criterion in the 3D-model, it is expected that more reliable results will be obtained if the researcher analyzes it in specific direction criteria and anatomy type.

Our study, taking into account risk factors such as gender, age, and smoking history, identified notable differences in hypertension, diabetes, and coronary artery obstructive disease correlating with the presence of aneurysms. Specifically, the AcomA group demonstrated significant variations in serum markers and blood lipid levels. These findings highlight the critical importance of considering patients’ medication histories to understand the impact of these conditions on aneurysm development fully.

Our study has several limitations. First, it relied on retrospectively collected data from a single-center database, which may limit generalizability. Future multicenter studies with larger cohorts are needed to improve data accuracy and robustness. The current analysis method requires manual designation of a reference point, potentially introducing variability, though efforts to refine and automate the algorithms are ongoing. The study did not evaluate the angles between A1, A2, and the anterior communicating artery due to measurement challenges. Given emerging evidence linking vascular angles and vessel tortuosity to aneurysm formation (12, 23), incorporating these factors in the future research could offer deeper insights into the relationship between vascular geometry and hemodynamic stress. The analysis focused on A1 asymmetry and AcomA formation, excluding other segments of the circle of Willis. Future investigations into regions such as the basilar artery, P1, posterior circulation bifurcations (24), and surrounding vessels of the COW (11) could provide complementary findings. Moreover, while we assessed hemodynamic trends by comparing relative shear stress between both sides, advancements in imaging techniques that allow rapid calculation of absolute shear stress values and flow direction analysis could enable more precise comparisons and a deeper understanding of hemodynamic mechanisms. Ultimately, analyzing the hemodynamic properties of the entire circle of Willis as a unified structure may offer a more holistic understanding of aneurysm formation and progression. Finally, classification of A1 aplasia relied solely on TOF-MRA, which may misidentify extreme asymmetry. Although we hypothesize that the hemodynamics of cases with extreme asymmetry likely resemble those of aplastic vessels (12, 20), including higher-resolution MRA or cerebral angiography would enhance diagnostic accuracy and better differentiate true aplasia from severe asymmetry. Future research should focus on confirmed cases analyzed with improved imaging techniques to provide a more comprehensive understanding of aneurysm formation.

## Conclusion

This study highlights the critical role of A1 segment asymmetry in anterior communicating artery aneurysm formation, emphasizing anatomical and hemodynamic factors. While the visualization of A1 shear stress via imaging is not yet superior to radius measurement alone, its integration with additional parameters, such as vascular angles, could provide a more comprehensive understanding of aneurysm mechanisms and contribute to improved prevention and management strategies.

## Data Availability

The raw data supporting the conclusions of this article will be made available by the authors, without undue reservation.
